# The Type of Anesthesia Used during Cesarean Section Is Related to the Transient Tachypnea of the Newborn

**DOI:** 10.1155/2013/264340

**Published:** 2013-04-24

**Authors:** Esengül Keleş, Hamza Yazgan, Arzu Gebeşçe, Emine Pakır

**Affiliations:** ^1^Department of Pediatrics, Fatih University, Turkey; ^2^Department of Family Medicine, Fatih University, Turkey

## Abstract

*Aim*. To demonstrate whether transient tachypnea of the newborn (TTN) is found more frequently in women undergoing general or combined epidural-spinal (CES) anesthesia during Cesarean section. *Methods*. This study was done retrospectively. A total of 1447 Cesarean sections (C/S) were performed in our clinic between January 2008 and December 2011. General anesthesia was performed in 1078 (74.5%) of the Cesarean cases. CES anesthesia was performed in 369 cases (25.5%). The International Classification of Diseases,Tenth Revision code of P22.1, was used to identify the infants with TTN. Stratified multivariate analysis was undertaken on subgroups to assess the effect modification by factors known to influence the incidence of TTN: maternal age, maternal systolic-diastolic artery pressure, heart rate, Apgar score at 1 and 5 minutes, sex, time interval from spinal block to skin incision, and time interval from skin incision to umbilical cord clamping. *Results*. The rate of TTN diagnosis was found to be higher in parturients who had a cesarean section with combined epidural-spinal anesthesia, but no statistical differences were found. (*P* < 0.05) (odds ratio = 1.471 and 95%CI: 0.92–2.35). *Conclusions*. The incidence of TTN was found related to C/S but independent from the type of anesthesia. However, studies with a wider spectrum of patients and a lower quantitative difference between the groups are needed in order to draw firm this conclusions.

## 1. Introduction

TTN is the most common cause of respiratory distress in newborns [1]. It is defined as respiratory distress thought to arise from a delay in fetal lung fluid absorption; it appears within the first six hours after delivery and resolves spontaneously with supportive therapy within a couple of days. The pathophysiology of transient tachypnea of the newborn (TTN) has not been fully explained. Potential factors include lack of exposure to the increased effect of catecholamines and other hormones in deliveries, which are initiated before the labor begins and insufficient activity of amiloride-sensitive epithelial sodium channels (ENaCs) [2–7]. Other potential risk factors such as negative amniotic fluid phosphatidylglycerol, Apgar score < 7, male gender, and failure to evacuate fetal lung fluid due to insufficient pressure in the thorax related to cesarean section (C/S) delivery have been reported for TTN [8].

Cesarean is derived from the Latin verb “caedere” meaning “to cut”. It was first used in 700 BC during the Roman era in order to remove a baby from the womb of a mother who died during childbirth during advanced stages of pregnancy. And the first C/S on a living patient was performed in 1610. While the safety and optimal conditions for one person are the prime concerns in a regular surgical intervention, the safety of the mother, the fetus, and the newborn should also be ensured in C/S. And this adds a new dimension to anesthesia for C/S. General and regional anesthesia techniques are used in anesthesia for C/S.

It is already a common knowledge that the incidence of TTN increases in C/S, but there is only a limited number of studies covering the effects of whether general or combined epidural-spinal anesthesia (CES) used in elective Cesarean deliveries on the incidence of TTN. The aim of the study was to determine whether the type of anesthesia used during Cesarean delivery is related to TTN.

## 2. Material and Method

A total of 1447 C/S were performed at the Sema Research and Training Hospital, Fatih University, between January 2008 and December 2011. 

The International Classification of Diseases, Tenth Revision code of P22.1, was used to identify the infants with TTN. Inclusion criteria were the following: a respiratory rate higher than 60 per minute within six hours after delivery, tachypnea lasting for at least 12 hours, increased aeration on chest X-ray, flattening of the ribs, fluid accumulation in the fissures and costophrenic angle, and vascular congestion and exclusion of either known respiratory (meconium aspiration, respiratory distress syndrome, pneumonitis, and congenital cardiac diseases) or nonrespiratory disorders (hypocalcaemia, persistent hypoglycemia, and polycythemia). Cesarean cases who were admitted to the newborn intensive care unit with a diagnosis of TTN were classified into two groups based on anesthesia techniques. The first group consisted of cases who received general anesthesia, whereas the second group consisted of cases who received CES anesthesia. 

Ineffective cases, plural pregnancies, preterm pregnancies, and newborns with fetal anomalies and fetal growth restriction were excluded from the study. Pregnant women with diabetes mellitus, hypertensive diseases, and asthma bronchioles that could affect acid-base balance were also excluded from the study.

Three hundred sixty parturients who underwent elective Cesarean section under CES anesthesia and were enrolled in this retrospective study received the same anesthesia and surgical care. Two hours before the implementation of the CES anesthesia technique, 1000 mL isotonic saline was infused via a venous catheter to all parturients in one hour. All women received aspiration prophylaxis with intravenous ranitidine 50 mg and metoclopramide 10 mg half an hour before the operation. Routine monitorization included heart rate, ECG, mean arterial pressure (MAP), and peripheral oxygen saturation. CES anesthesia was performed in the sitting position according to the general rules of asepsis and antisepsis. L3-L4 or L4-L5 interspaces were identified with reference to the iliac crest. Spinal anesthesia was performed with a 25 G pencil point spinal needle using hyperbaric bupivacaine 10 mg + fentanyl 10 mg in the sitting position by midline approach. Hypotension which had been defined by more than 20% decrease from baseline MAP was treated with 10 mg dose of intravenous bolus ephedrine. 

In general anesthesia, propofol of 2 mg/kg was administered. Once the patient was intubated, a mixture of 50% NO_2_ and 50% O_2_ was administered by inhalation.

### 2.1. Statistical Analysis

The NCSS (Number Cruncher Statistical System), 2007, and PASS (Power Analysis and Sample Size), 2008 (Utah, USA), statistical software were used to analyze the findings of this study for statistical analyses. When analyzing the study data in addition to descriptive statistical methods (average, standard deviation, median, frequency, and ratio), Student's *t*-test was used in normally distributed parameters, and Mann-Whitney *U* test was used in non-normally distributed parameters to compare quantitative data between the two groups. And the Pearson's chi-square test and the Yates' corrected chi-square test (Yates continuity correction) were used to compare qualitative data. The results were evaluated in a confidence interval of 95% and at a significance level of *P* < 0.05.

## 3. Results

General anesthesia was performed in 1078 (74.5%) of the Cesarean cases (group 1), and CES anesthesia was performed in 369 cases (25.5%) (group 2). We examined a total of 85 (5.87%) term infants treated in the NICU for TTN.  The maternal ages of the 85 cases being followed up at the NICU for TTN ranged between 20 and 44, and the average age was 32.25 ± 4.39 years in group 1 and 32.18 ± 5.27 in group 2; in group 1, 73.7% were male and 26.3% were female, and, in group 2, 67.9% were male and 32.1% were female; average hospitalization at the NICU was 4.04 ± 2.74 days in group 1 and 3.57 ± 2.51 days in group 2 with no statistically significant differences between both groups. Average systolic and diastolic blood pressure was 115.13 ± 18, 76.3 ± 7.9 mmHg in group 1 and 104.09 ± 12, 68.4 ± 8,7 mmHg in group 2; the average pulse rate was 78.3 ± 5.1 pulses/min in group 1 and 76.2 ± 7.1 pulses/min in group 2 with no statistically significant differences between both groups (Table 1).

One-hour postnatal pH value in blood gases was 7.3 ± 0.02 in group 1 and 7.3 ± 0.04 in group 2; PCO_2_ value was 49.0 ± 4.3 in Group 1 and 47.8 ± 3.7 in Group 2; PO_2_ value was 56.4 ± 2.0 in Group 1 and 57.0 ± 3.6 in Group 2; Apgar scores at 1 minute were 6.1 ± 1.0 for Group 1 and 6.5 ± 0.9 for Group 2; Apgar scores at 5 minutes were 8.3 ± 0.7 for Group 1 and 8.5 ± 0.8 for Group 2 with no statistically significant differences between both groups. All spinal anesthesia procedures were performed by the same anesthesia specialist in all cases. The mean time interval from spinal block to skin incision was 8.2 ± 2.93 minutes. About 80% of the Cesarean section deliveries were performed by the same gynecologist. The mean time interval from skin incision to umbilical cord clamping was 2.69 ± 1.8 minutes in Group 1 and 2.48 ± 1.3 minutes in Group 2. No statistically significant differences were found between both groups (*P* < 0.1) (Table 1). 

General anesthesia was performed in 57 (5.3%), and CSE anesthesia was performed in 28 (7.6%) of the TTN cases (Table 2). Although this rate was found higher in cases who had CSE anesthesia, since the number of cases was 3.9 times more in those cases who had general anesthesia, no statistical differences were found between TTN incidence rates by the type of anesthesia used (*P* < 0.105).

In the group receiving spinal anesthesia, 6% of the cases had nausea, 5% vomiting, 4% sedation, 53% trembling, and 7.6% TTN.

## 4. Discussion

No anesthetic agent or technique is ideal for parturients. The normal spontaneous vaginal delivery (NSVD) should always be the primary choice since it is more physiological. However, in special cases where NSVD may not be applicable, the choice of anesthesia depends on the cause of the intervention, its degree of emergency, the patient's demand, obstetric requirements, and the expertise of the anesthetist. The anesthetist must select a method that is the safest and most comfortable for the mother and that ensures the least depressing working conditions for the newborn and the optimal working conditions for the surgery. General and regional anesthesia techniques are used in anesthesia for Cesarean delivery.

General anesthesia offers advantages in situations where there is a race against time including fetal distress, prolapse of cord, placenta previa, or the baby's arm coming out first because of the fast induction and in situations where regional anesthesia is contraindicated including coagulopathy, infections, and bleeding as well as where the patient who has no other contraindications disapproves regional methods. It may also be considered advantageous due to lower risks of hypotension, better maintenance of cardiovascular stability, and improved control of the airway and ventilation. And the risks of general anesthesia are the pulmonary aspiration of gastric contents and difficulty with intubation [2–4].

Regional anesthesia is described as the removal of neural conduction and the pain sensation in particular regions of the body without causing any loss of consciousness. Dramatic analgesia was first obtained through epidural and intrathecal opioid applications in 1979. Regional anesthesia is more frequently preferred in recent years due to its advantages including the patient's demand, conscious patient, no risks of aspiration, no respiratory depression in newborns, and no uterine atony. Its side effects in the mother include neurotoxicity, hypotension, nausea, vomiting, respiratory depression, urinary retention, delayed gastric emptying, postdural headache, total spinal block, anterior spinal artery syndrome, back and belly aches, and hypothermia. And fetal side effects develop depending on the systemic absorption of epidural opioids or their side effects on the mother [5].

CES anesthesia may reduce some of the disadvantages of spinal and epidural anesthesia while maintaining its advantages. CES anesthesia ensures that the period of anesthesia provided by epidural anesthesia can be extended by the rapid onset, efficiency, and minimal toxic effect produced by the spinal block. CES anesthesia causes minimal block because the anesthesia technique allows drug titration. It was demonstrated to be more reliable as it provided selective sensory blockade.

The most important cause of fetal distress in both anesthetic methods is the reduction in the amount of O_2_ available to the fetus as a result of the reduction of uteroplacental blood flow. Maternal, placental, and fetal factors play roles in such reduction. The effect of anesthetic drugs is direct or through the changes in the mother [6].

When the induction-delivery interval exceeds 10 minutes in general anesthesia, fetal tissues are saturated with NO_2_. As a result, a mild depression and, if oxygenation is not sufficient, diffusion hypoxia can occur in the newborns during the first minutes. About 80% of the Cesarean section deliveries were performed by the same gynecologist at our hospital. And this resulted in standardized induction-delivery intervals. This interval was found to be three minutes on average for both groups.

Bupivacaine is a commonly used agent during deliveries as it has a long-lasting effect, it is more markedly selective over sensory neural fibers than to motor fibers, and it has a lower fetal-maternal blood ratio [7]. When continuously infused epidurally, epidural opioids rarely cause respiratory depression. There are no significant differences between the Apgar score and sensory behavioral scores and the scores of the babies of mothers who took no medications. This is generally the temporary and may be associated with hypotension. At our hospital, 1000 mL physiological saline was infused within one hour preoperatively to all cases who received CES anesthesia in order to make sure no hypotension occurs in the mother. The use of 10 mg bupivacaine + 10 mg fentanyl was administered in all cases at our hospital during CES anesthesia. The performance of all spinal anesthesia procedures by the same anesthesia provider ensured a standardized mean time interval from spinal block to skin incision. This is because some studies in the literature reported that reducing the time interval from spinal block to the onset of surgery might be one of the important measures to decrease the incidence of TTN after elective Cesarean section under spinal anesthesia [9].

It is already a common knowledge that Cesarean surgeries are an important risk factor in the etiology of TTN. In our study, we planned to investigate the relationship between the type of anesthesia during the surgery and TTN independent from Cesarean application. Although our study found higher rates in cases who received CES anesthesia, since the number of cases was 3.9 times more in those cases who had general anesthesia, no statistical differences were found between TTN incidence rates by the type of anesthesia used. In conclusion, the incidence of TTN was found related to C/S but independent from the type of anesthesia. However, studies with a wider spectrum of patients and a lower quantitative difference between the groups are needed in order to be able to underline this conclusion. 

## Figures and Tables

**Table 1:** Demographics and clinical data according to general anesthesia versus combined epidural-spinal anesthesia.

	General anesthesia	Spinal anesthesia	*P *
	*n* = 57	*n* = 28	
	Median ± SD	Median ± SD	
Mother's age	32.25 ± 4.39	32.18 ± 5.27	0.951
Mother's:			
Systolic artery pressure mmHg	115.13 ± 18	104.09 ± 12	0.234
Diastolic artery pressure	76.3 ± 7.9	68.4 ± 8.7	0.136
Number of pulses (pulses/min)	78.3 ± 5.1	76.2 ± 7.1	0.386
Sex			
Male	42 (73.7%)	19 (67.9%)	0.761
Female	15 (26.3%)	9 (32.1%)	
NICUstay (days)	4.04 ± 2.74	3.57 ± 2.51	0.360
Apgar			
Minute one	8.3 ± 0.7	8.5 ± 0.8	0.633
Minute five	6.1 ± 1.0	6.5 ± 0.9	0.265
Blood gas			
CO_2_ (%)	49.0 ± 4.3	47.8 ± 3.7	0.236
O_2_	56.4 ± 2.0	57.0 ± 3.6	0.536
pH	7.3 ± 0.02	7.3 ± 0.04	0.445
Time interval from skin incision to umbilical cord clamping (min)	2.69 ± 1.8	2.48 ± 1.3	0.1
Time interval from spinal block to skin incision (min)		8.2 ± 2.93	

Student's *t*-test

Mann-Whitney *U* test

Continuity correction chi-square test.

**Table 2:** Evaluation of transient tachypnea of the newborn by type of anesthesia.

Transient tachypnea of the newborn	General anesthesia (*n* = 1078)	Spinal anesthesia (*n* = 369)	*P *
	*n* (%)	*n* (%)	
Exists	57 (5.3%)	28 (7.6%)	*0.105 *
None	1021 (94.7%)	341 (92.4%)	

Pearson's chi-square test
